# Regression of Atherosclerosis Is Characterized by Broad Changes in the Plaque Macrophage Transcriptome

**DOI:** 10.1371/journal.pone.0039790

**Published:** 2012-06-27

**Authors:** Jonathan E. Feig, Yuliya Vengrenyuk, Vladimir Reiser, Chaowei Wu, Alexander Statnikov, Constantin F. Aliferis, Michael J. Garabedian, Edward A. Fisher, Oscar Puig

**Affiliations:** 1 Division of Cardiology, and the Marc and Ruti Bell Program in Vascular Biology, Department of Medicine, New York University School of Medicine, New York, New York, United States of America; 2 Department of Cardiovascular Diseases, Merck Research Laboratories, Rahway, New Jersey, United States of America; 3 Department of Microbiology, New York University School of Medicine, New York, New York, United States of America; 4 Center for Health Informatics and Bioinformatics, New York University School of Medicine, New York, New York, United States of America; 5 Department of Medicine, Division of Translational Medicine, New York University School of Medicine, New York, New York, United States of America; 6 Department of Pathology, New York University School of Medicine, New York, New York, United States of America; 7 Informatics and Analysis, Merck Research Laboratories, Kenilworth, New Jersey, United States of America; Centro Cardiologico Monzino IRCCS, Italy

## Abstract

We have developed a mouse model of atherosclerotic plaque regression in which an atherosclerotic aortic arch from a hyperlipidemic donor is transplanted into a normolipidemic recipient, resulting in rapid elimination of cholesterol and monocyte-derived macrophage cells (CD68+) from transplanted vessel walls. To gain a comprehensive view of the differences in gene expression patterns in macrophages associated with regressing compared with progressing atherosclerotic plaque, we compared mRNA expression patterns in CD68+ macrophages extracted from plaque in aortic aches transplanted into normolipidemic or into hyperlipidemic recipients. In CD68+ cells from regressing plaque we observed that genes associated with the contractile apparatus responsible for cellular movement (e.g. actin and myosin) were up-regulated whereas genes related to cell adhesion (e.g. cadherins, vinculin) were down-regulated. In addition, CD68+ cells from regressing plaque were characterized by enhanced expression of genes associated with an anti-inflammatory M2 macrophage phenotype, including arginase I, CD163 and the C-lectin receptor. Our analysis suggests that in regressing plaque CD68+ cells preferentially express genes that reduce cellular adhesion, enhance cellular motility, and overall act to suppress inflammation.

## Introduction

Cardiovascular disease (CVD) is a leading cause of death worldwide [1]. Absolute risk for CVD rises with age because of the progression of atherosclerosis [2]–[5]. This risk could be reduced by blocking the progression of atherosclerosis or by stimulating regression of atherosclerotic plaque. Mouse models of atherosclerosis, particularly lines with deficiencies in apoE (apoE−/−) or the LDL receptor (LDLr−/−) [6]–[8], have been extensively used to study atherosclerotic progression and for the identification of therapeutic approaches to blocking it. However, application of these models to the study of plaque regression has not been as actively pursued.

Our laboratory has developed a model of plaque regression in which an aortic arch segment containing an atherosclerotic plaque from an apoE−/− mouse [9] is transplanted to a wild-type (WT) recipient mouse. This results in the rapid normalization of the dyslipidemia to which donor plaques are exposed and a rapid regression of atherosclerosis as judged by a decrease in both plaque size (i.e., cross sectional intimal area) and the content of monocyte-derived CD68+ cells (primarily macrophages and macrophage foam cells). In contrast, when the recipient of the atherosclerotic arch is an apoE−/− mouse and the dyslipidemia is not corrected, progression continues [10]–[12]. Using laser capture microdissection to select plaque CD68+ cells under progression and regression conditions, and qPCR to analyze selected mRNAs, we found large differences in the expression of specific genes involved in migration and inflammation [12], [13].

In the current study a transcriptome wide analysis of the macrophage-specific changes associated with plaque regression was performed. Microarray assays of mRNA from laser captured CD68+ cells revealed notably different molecular profiles in cells from plaques in atherosclerotic aortic arches transplanted into apoE−/− (progression environment) compared with those from plaques in arches transplanted into WT recipients (regression environment).

## Methods

### Animals and Aortic Transplantation

This study was performed in strict accordance with the recommendations in the Guide for the Care and Use of Laboratory Animals of the National Institutes of Health. The protocol was approved by the Institutional Animal Care and Use Committee of the New York University School of Medicine (Permit Number: A3435-01). ApoE−/− (C57Bl/6) mice were weaned at 1 month and put on a Western Diet (WD) containing 21% fat and 0.15% cholesterol (Research Diets catalog N D01022601) for 16 weeks. These mice were then divided into three groups: a pre-transplant group (*n* = 10) for baseline analyses, and two transplant groups, a group (*n* = 18) from which aortas would be transplanted into apoE−/− recipient mice, and a group (*n* = 18) from which aortas would be transplanted into wild type recipient mice. In both cases, recipients were 20 week old male mice on standard chow diet. The surgeries were performed as previously described [9]. Briefly, a donor arch was interpositioned with the abdominal aorta in the recipient mouse and blood flow was directed through the graft. Recipient mice were maintained on standard chow diet, and sacrificed at 3 days after transplantation.

### Lipid and Lipoprotein Analyses

Blood samples were obtained from the retro-orbital plexus. Plasma total cholesterol and triglyceride levels were determined by colorimetric enzymatic assays adapted to 96-well plate formats (Infinity Total Cholesterol Reagent or Infinity Triglyceride Reagent, Sigma). Plasma HDL cholesterol was determined by precipitating non-HDL cholesterol (Wako Diagnostic) and then assaying the remaining HDL cholesterol by means of the Infinity Total Cholesterol Reagent.

### Lesion Assessment by Immunostaining, Histology and Morphometry

The pre-transplant and grafted arches were removed, embedded in OCT, and frozen. Serial sections (6 µm thick) were generated using a cryostat. To detect monocyte-derived cells (predominately macrophages and macrophage foam cells), sections were stained for CD68 using rat anti-mouse CD68 (Serotec; 2 µg/ml). Briefly, sections were incubated for 1 hour at room temperature with the primary antibody, then with biotinylated goat anti-rat Ig for 1 hour, followed by incubation with streptavidin-linked alkaline phosphatase, then developed with substrate, and finally counterstained with haematoxylin. Negative controls were performed with an irrelevant primary antibody. To determine the plaque area, images were digitized, the regions between the endothelial and the medial smooth muscle cell layers were traced, and ImagePro Plus software was used to perform the numerical calculations. A similar approach was taken for the area stained for CD68 or Oil Red O. At least five sections per vessel were analyzed and the mean value used as the summary parameter. The same immunostaining protocol with different primary antibodies was used in order to detect arginase I (Santa Cruz Biotechnology, sc-20150) or vinculin (Abcam, ab18058). To assess the lipid content of the plaques, Oil Red O staining was performed as previously described [14]. This stain will detect all neutral lipids, the predominant species being cholesteryl esters and triglycerides. In plaques, traditionally Oil Red O staining is thought to reflect the intra and extracellular content of cholesteryl esters, though it is known that triglycerides are also found in foam cells to a variable degree.

### Laser Capture Microdissection (LCM) and RNA Extraction

To isolate CD68+ cells from plaques, LCM was performed with the PixCell II (Arcturus Bioscience, Mountain View, CA) as previously reported [15]. Briefly, 6 µm frozen sections were dehydrated in ethanol, then twice placed in xylene and air dried. At 100 µm intervals, sections were immunostained for CD68 and used as templates for the next five serial sections. RNA was isolated by the Qiagen RNeasy MicroIsolation kit and treated with DNase. The concentration of RNA was determined by the Ribogreen RNA Quantification kit (Molecular Probes), and the RNA quality verified with the Agilent 2100 Bioanalyzer. Each microarray RNA sample from laser-captured CD68+ cells represented a pool of LCM-derived RNA from three aortas, with an average total RNA per sample of 5 ng. Approximately 2000 CD68+ cells are obtained from each aorta.

### RNA Processing and Microarray Analysis

Two rounds of amplification with an ultra low input protocol (ULI) were used to generate cRNA labeled with Cy5 or Cy3 from sample RNA (∼1–5 ng) generated by LCM [16]. This cRNA was combined with an equal mass of universal mouse reference RNA that had been amplified in the same way but labeled with the opposite fluoro. This mixture was then hybridized to Mouse 3.0 A1 Agilent arrays containing 23427 reporters (not including control probes, Gene Expression Omnibus (GEO) accession GPL9733). To avoid bias from the Cy3 and Cy5 labels, fluoro reverse duplicates were used for all samples (each sample was labeled independently with Cy3 and Cy5, mixed with labeled control pool RNA, and each reaction was independently hybridized to a single microarray, therefore using two arrays per sample). Data were pre-processed using the standard re-ratio pipeline in Rosetta Resolver application [17]. Samples were processed in two independent batches (with four samples from each of the three groups in each batch). To normalize for batch to batch variation, samples were first re-ratioed against the pool of all samples in each batch. Regression-specific genes, as identified by univariate analysis, were defined by two criteria: Regulated by regression (Progression vs. Regression ANOVA p<0.05) and not regulated by transplantation (Progression vs. Baseline control ANOVA p>0.1). To select genes with stable variance, a final filter using Resolver error model P<0.05 was applied [17], and to select for larger effect size a 1.2 fold change threshold was applied. K-means clustering (K = 2) was used to separate transcripts upregulated from transcripts down-regulated in regression conditions. The proportion of false positives was estimated at 26% by permuting 100 times the labels in each dataset prior to any gene filtering, calculating the size of the differential profile in each permutation, and averaging the results to determine the level of noise. Raw data are available in GEO (accession number GSE24819). Pathway analysis was performed using the Ingenuity Pathway Analysis tool (MIPA) (Ingenuity Systems, Redwood City, California) or GeneGo Metacore (GeneGo, Inc., St. Joseph, Michigan). Enrichment for biological processes was performed as described [18]. Briefly the genes in the differential profiles were compared to gene families in Gene Ontology, KEGG, Swissprot and Panther databases. P values (Fisher's exact test) were corrected for multiple testing (Bonferroni correction, 0.05 alpha-level).

### De novo Local Causal Pathway Discovery

To discover de novo causal pathways of plaque regression and select genes for predictive molecular signatures, we used a multivariate gene selection technique (termed Semi-Interleaved HITON-PC without symmetry correction). HITON-PC was applied with Fisher's Z-test, α = 0.05 and *max-k = *1 [19], [20]. To identify predictive signatures of regression from selected genes, we used linear support vector machine (SVM) classifiers with default value of *C* = 1. We used leave-one-out cross-validation (LOOCV) approach to estimate predictive accuracy (area under ROC curve) of molecular signatures. The statistical significance of the produced multivariate signatures was tested with a previously developed protocol for assessing statistical significance of multivariate signatures at 0.05 alpha-level [21].

### Quantitative Real Time PCR (qPCR)

qPCR was used to validate specific genes of interest using 100 pg of total RNA per reaction. The primer and probe sequences are shown in Table S1. All data were normalized to cyclophilin A or GAPDH and expressed as fold change from the controls. Two independent biological replicates, each one consisting of RNA from a pool of CD68+ cells from three aortas, were used to validate each transcript by qPCR. Data were expressed as mean ± SEM and were analyzed by one-way ANOVA. P<0.05 was considered significant.

## Results

### Normalization of dyslipidemia decreases CD68+ cell and cholesteryl ester contents in atherosclerotic plaque

**Figure 1:**
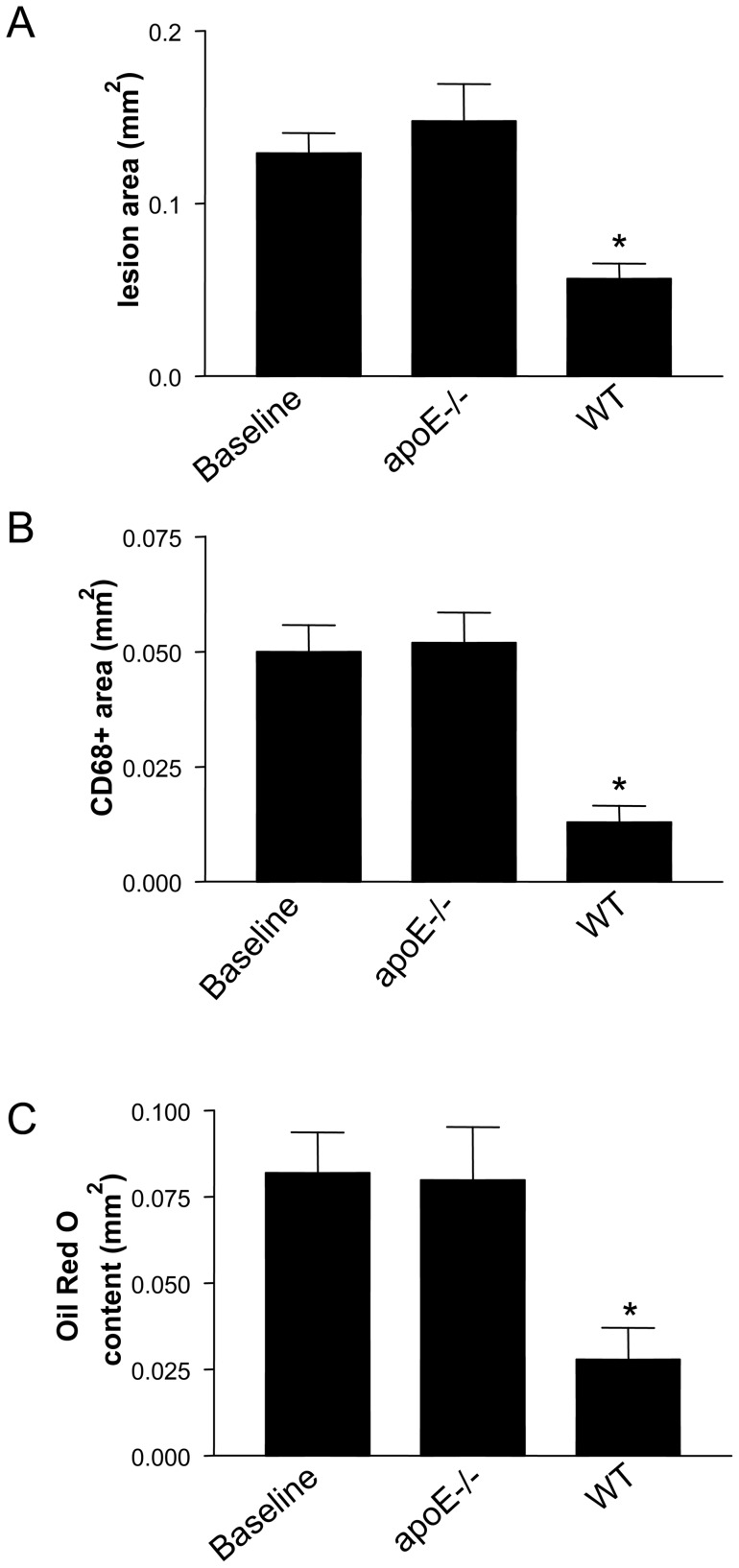
Transfer of plaques to a normolipidemic plasma environment decreases their size and reduces their burden of CD68+ cells and cholesteryl esters. Transplantation experiments were performed as described in Methods. Reversal of dyslipidemia (WT recipient) decreases plaque size (A) and CD68+ cell content (B) but continued dyslipidemia (apoE−/−) does not. Cholesteryl ester content was estimated by Oil Red O staining, which showed a decrease when the recipient was a WT mouse but not an apoE−/− mouse (C). The symbol * corresponds to statistical significance, p<0.05, when compared to donor apoE−/− mice (Baseline).

ApoE−/− mice were fed a high fat diet for 16 weeks (“baseline mice”) after which the aortic arches were transplanted into either apoE−/− (progression environment) or WT mice (regression environment) [12]. Three days after transplantation, mice were sacrificed, and plasma and transplanted aortic arches were harvested. There was a 57% reduction in plaque size (Figure 1A), and a 62% reduction in CD68+ cell content (Figure 1B) when the recipients were WT compared to the apoE−/− recipients. In addition, the lipid content, most likely cholesteryl ester, decreased significantly only under regression conditions, as measured by Oil Red O staining (Figure 1C). The mean total plasma cholesterol level in the wild type mice was 10% of that in donor apoE−/− mice, whereas the mean HDL level in the WT recipients was approximately three times higher than in donor apoE−/− mice (Table 1).

**Table 1:** Summary of lipoprotein profile of apoE−/− donors, WT recipients, and apoE−/− recipients. Values are mean ± SD of 11 mice per group.

Groups	HDL-C, mg/dL	Total Cholesterol, mg/dL
apoE−/− Donors	21±3	1010±203
apoE−/− Recipients	24±4	562±41*
WT Recipients	69±9**	106±12**

* P<0.05 **P<0.0001.

### Regression of atherosclerosis is associated with large-scale change in the CD68+ macrophage transcriptome

**Figure 2:**
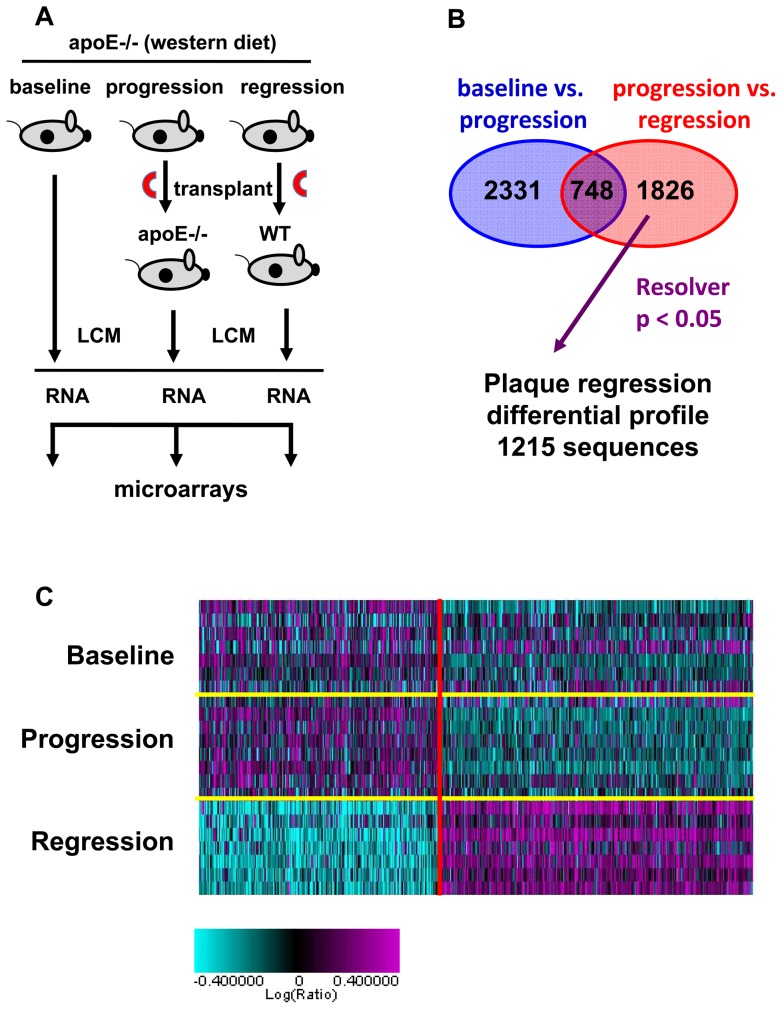
Identification of a plaque regression differential profile. (A) Depiction of the procedure used to determine the plaque regression differential profile. Plaques from apoE−/− mice on a high fat diet were left intact (baseline) or the aortic arch (red semi circle) was transplanted into WT (regression) or apoE−/− (progression) mice. After 3 days, CD68+ cells were selected by Laser Capture Microdissection (LCM), RNA was prepared, amplified, labeled and hybridized to DNA microarray containing 23623 sequences. (B) Sequences affected by the transplantation procedure were defined as genes that were different in baseline (not transplanted) vs. progression (transplanted) groups (ANOVA P>0.1) and were removed from analysis. This left 1826 sequences that were differentially expressed between progression and regression groups (ANOVA P<0.05, in red). This set was further refined using Resolver error model (P>0.05), which eliminates unreliable variance estimation, and a 1.2 fold change filter, resulting in 1215 sequences that are referred to as the “plaque regression” differential profile. C) Heat map of genes differentially expressed in baseline, progression and regression. Individual aortic samples are on the Y-axis, individual genes are on the X-axis.

To gain a deeper understanding of the molecular pathways involved in plaque regression, we compared the gene expression profiles in plaque CD68+ cells from 1) baseline apoE−/− mice (i.e., the aortic arch donors), 2) progressing mice (apoE−/− donors to apoE−/− recipients) and 3) regressing mice (apoE−/− donors to WT recipients) using microarray technology (Figure 2A). First, transcripts affected by the transplantation procedure rather than by regression *per se* were identified as those genes that were differentially expressed in baseline (not transplanted) vs. progression (transplanted) groups, and were removed from the analysis. Then transcripts affected by regression were identified as those differentially expressed in the progression vs. regression group. A fold change of 1.2 was used as a threshold to define 1215 transcripts as the “plaque regression” differential profile (Figure 2B, 2C and Table S2).

**Figure 3:**
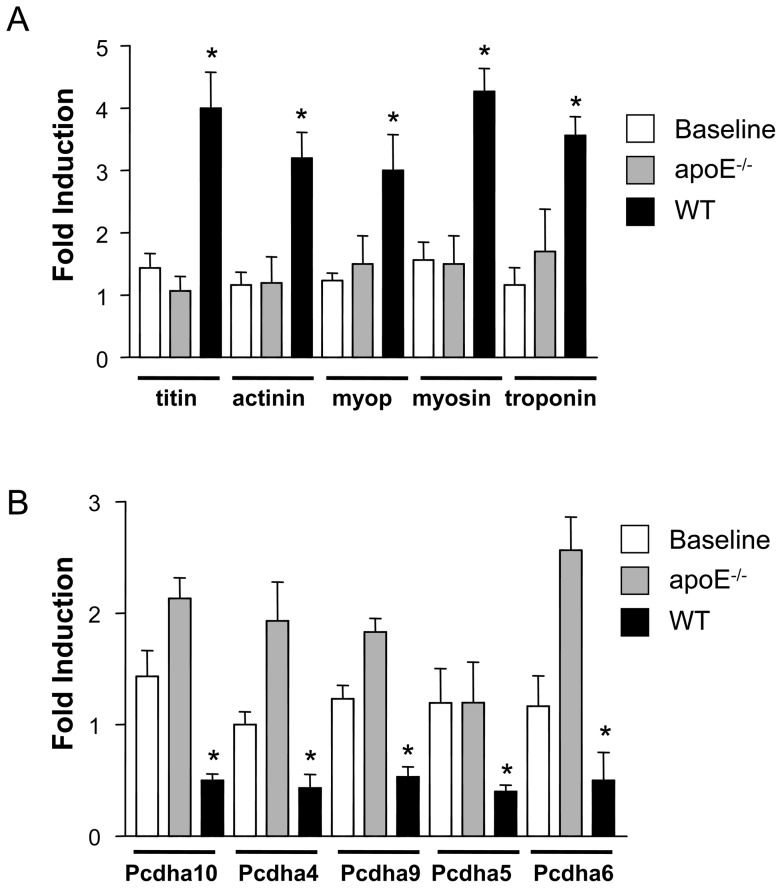
Regression is characterized by increasing expression of contractile proteins and down-regulation of cadherins. CD68+ cells were selected from plaques by LCM, RNA was isolated and qPCR performed in order to validate microarray results. The results of qPCR showed that the contractile proteins were up-regulated (A), whereas members of the cadherin family were down-regulated (B). The symbol * corresponds to statistical significance, p<0.05, when mRNA level in the aortas from a transplanted group (apoE−/−, WT) was compared to mRNA in aortas from donor apoE−/− mice (Baseline). 9 mice were analyzed per group. Cyclophilin A was used for normalization in both cases.

We determined which biological processes were enriched in the plaque regression differential profile by comparing this gene set with gene sets classified by biological function in the public gene set collections Gene Ontology, KEGG, Swissprot and Panther families. This analysis revealed that up-regulated transcripts in the plaque regression differential profile were enriched for contractile proteins (Tables S3 and S4). The top three biological processes enriched in genes up-regulated in regression vs. progression were “sarcomere”, “myofibril” and “muscle contraction”. Transcripts down-regulated in the plaque regression differential profile were enriched for “protocadherin” family members, which are proteins that promote cellular adhesion, and for cell cycle/cell division gene families (“DNA metabolism”, “histone”, “cell cycle”, and “DNA replication”) (Tables S3 and S4). To validate microarray data we performed qPCR. We confirmed up-regulation of several genes associated with the contraction (titin, α-actinin, myopalladin, myosin heavy chain, and troponin) in WT recipients (Figure 3A). We also confirmed the down-regulation of protocadherins 4, 5, 6, 9 and 10 under regression conditions (Figure 3B).

**Figure 4:**
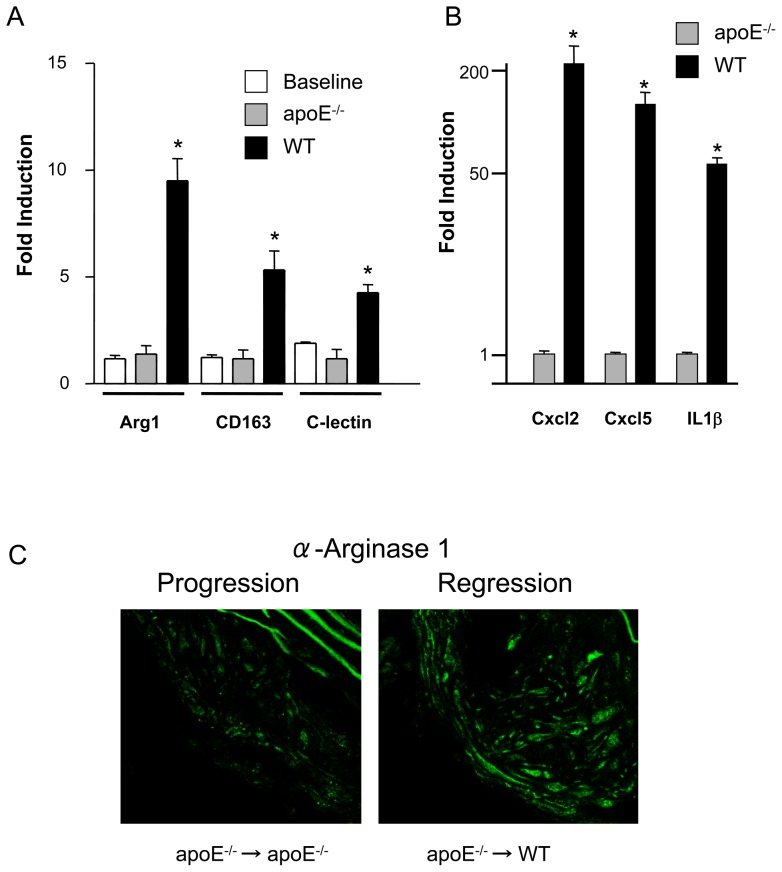
Regression is characterized by macrophages expressing M2 markers. (A) qPCR analysis for RNA encoding arginase I (Arg I), CD163 and C-lectin receptor was performed on RNA extracted from CD68+ cells removed from plaques that were not transplanted (baseline), plaques transplanted into apoE−/− recipients (progression environment) and plaques transplanted into wild type recipients (WT, regression environment). The symbol * corresponds to statistical significance, p<0.05, when signal in RNA from transplanted aortas (apoE−/−, WT) was compared to signal in RNA from aortas in donor apoE−/− mice (Baseline). 9 mice were analyzed per group. Cyclophilin A was used for normalization. (B), qPCR analysis for RNA encoding Cxcl2, Cxcl5 and IL1b. The symbol * corresponds to statistical significance, p<0.05, when signal in RNA from apoE−/− was compared to signal in RNA from WT mice. 9 mice were analyzed per group. GAPDH was used for normalization. (C) Arginase I staining shows increased protein levels in regression.

The most highly up-regulated gene (14 fold) under regression conditions was arginase 1. Up-regulation of this gene was validated by qPCR (Figure 4A) and at the protein level (Figure 4C). Up-regulation of some inflammatory markers (Cxcl2, Cxcl5 and IL1β) was confirmed by qPCR (Figure 4B). Because arginase 1 is considered a classical marker of the M2 (anti-inflammatory or tissue repair) state of macrophages [22], we determined whether other accepted M2 markers are present in the plaque regression differential profile. We found that CD163 and C-lectin receptor gene expression were also upregulated in the microarray results (Table S2) and when evaluated by qPCR (Figure 4A).

### Local causal pathway discovery analysis

**Figure 5:**
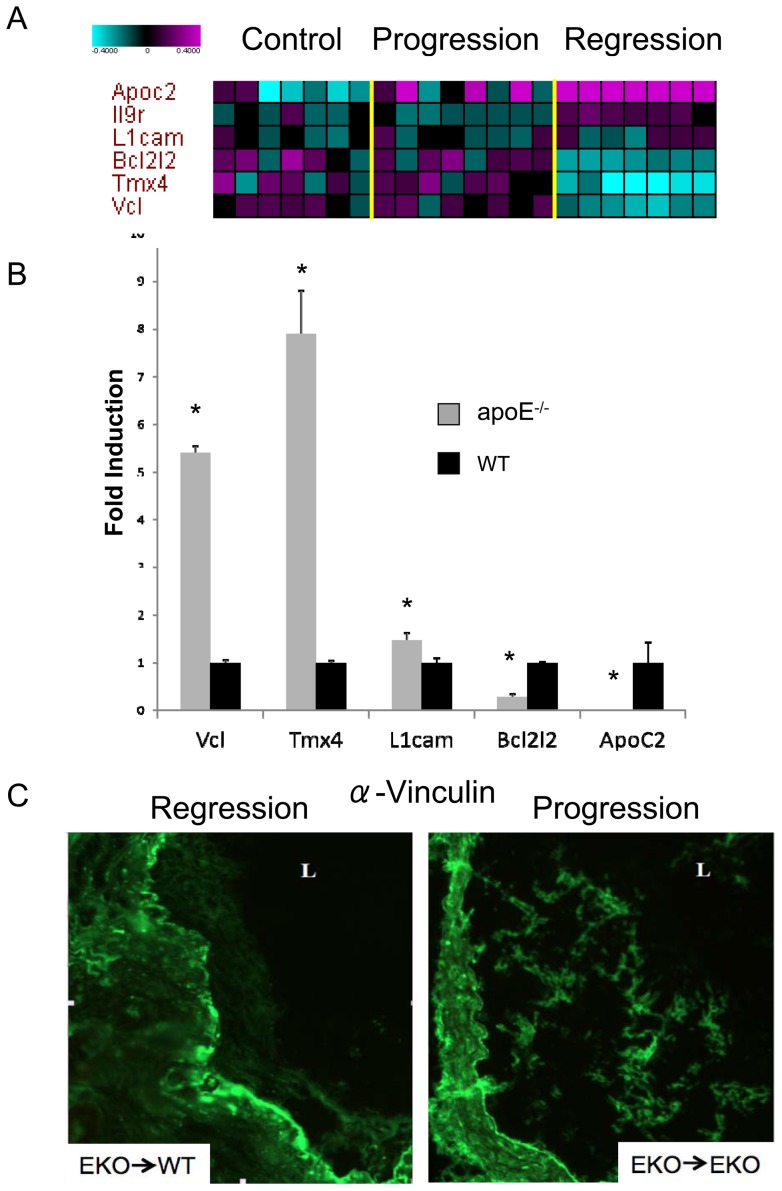
Multivariate causal pathway analysis for genes involved in regression. (A) Heatmap displaying the relative levels of the 6 genes selected by HITON-PC algorithm for causal relationship to regression vs. progression/baseline task (Y-axis) in each of the 22 analyzed aortic samples (X-axis). B) RNA was isolated from laser captured CD68+ cells. qPCR was then performed in order to validate microarray results. The symbol * corresponds to statistical significance, p<0.05, when signal in RNA from aortas in regression (apoE −/−) was compared to signal in RNA from aortas in progression (WT). 9 mice were analyzed per group. GAPDH was used for normalization. C) Immunostaining for vinculin in a plaque derived from an aortic arch transplanted from an apoE−/− mouse into a wild type normolipidemic mouse (apoE−/− > WT) and from an apoE−/− mouse into a different apoE−/− mouse (apoE−/− > apoE−/−).

To identify genes that may play a causal role in atherosclerotic plaque regression, we applied a multivariate causal pathway discovery method called HITON-PC, and then used these genes to build multivariate molecular signatures to predict regression and to estimate the accuracy of their predictive capability. This approach distills a large global data set signature (based on a univariate model, including all genes that are differentially expressed in each experimental group) into a smaller local cause and effect predictive signature (based on a multivariate model, including only those genes which are directly upstream and downstream from the response variable of interest) [19], [20]. We focused on building a multivariate molecular signature for regression group vs. baseline and progression groups together, since this is the most stringent comparison to reveal genes with the potential for “driving” regression. This approach identified six genes in the local causal pathway of regression, three down regulated in the regression environment (Vcl, Tmx4, Bcl2l2) and three upregulated (IL-9r, ApoC2 and L1cam) (Figure 5A) and achieved very good predictive accuracy (0.838 area under the curve (AUC) and p = 0.004). We used qPCR to validate changes in several of these factors in regression vs. progression, and Vcl, Tmx4 and ApoC2 were validated (Figure 5B and data not shown). Validation by protein expression for Vcl (vinculin-1) was done by immunohistochemistry to confirm that the gene product was also down regulated (Figure 5C).

## Discussion

We have determined a molecular profile of atherosclerosis regression in a mouse transplant model by performing microarray analysis of plaque CD68+ cells. The profile consisted of 1215 transcripts that were differentially expressed, indicating a large change in the transcriptome of CD68+ cells between the regression and progression environments.

The plaque regression differential profile revealed a number of findings: 1) genes associated with the contractile apparatus, which plays a crucial role in cellular movement were among the most highly up-regulated; 2) genes encoding proteins that mediate cellular adhesion, like cadherins and vinculin, were significantly down-regulated; 3) genes encoding histones, cell cycle, cell division and DNA metabolism were also significantly down-regulated, suggesting inhibition of cell division during regression; 4) during regression macrophages display an M2-like state with increased expression of classical markers such as arginase I, CD163 and the C lectin receptor. The last findings led to independent studies that have shown enrichment of M2 macrophage factors in other models of regressing plaques [13], [23].

The process that stimulates the elimination of CD68+ cells from regressing plaques has not been fully defined. Our previous studies indicate an important functional role for CCR7 in regulating CD68+ emigration process during regression [12]. In addition, our microarray analysis revealed further activities related to cell emigration during regression. For example, in CD68+ cells in the regression environment, we observed that the contractile machinery is up-regulated, which would allow for the cells to migrate out of the lesion. It is unlikely the appearance of contractile genes in the plaque regression differential profile is due to contamination of macrophages with smooth muscle cells, since our previous studies have shown a large (>30 fold) and significant enrichment in macrophage-specific transcripts relative to smooth muscle cell mRNAs from laser captured CD68+ cells [15]. We and others have also shown that under conditions of atherosclerosis progression, proteins such as VCAM-1 and ICAM-1 are up-regulated [24]–[26]. These proteins, and the cadherins that we identified in the present study, are cell-cell adhesion factors that help cells maintain their location. Under regression conditions, however, these genes are down-regulated, which would allow for the cells to “detach” and begin their exit from the plaque. Thus, our findings indicate that the emigration process underlying atherosclerosis regression reflects a complex, yet highly organized series of events that results in decreased cellular retention and increased contractile events that together promote efficient cell migration. Part of the complexity of cellular emigration may also be reflected by the decrease in levels of mRNAs that encode factors involved with cell division observed in the plaque regression differential profile, suggesting that cell division may cease when the priority of the cell is to migrate. Observed changes in mRNAs encoding chromatin remodeling factors may indicate large-scale alterations in the epigenetic control of gene expression.

There is macrophage heterogeneity in atherosclerotic plaques, both M1 (inflammatory) and M2 (anti-inflammatory) macrophages are detectable in lesions [27]. We observed M2 markers are up-regulated under regression conditions. In fact, the establishment of an anti-inflammatory state in plaque CD68+ cells during regression appears to be a general finding [13], [23]. Whether in regressing plaques this reflects a switch from a M1 to M2 phenotype, or the replacement of M1 with M2 macrophages is currently under investigation.

Our local causal pathway discovery analysis revealed a molecular signature that could distinguish between the treatment groups with high accuracy. Several genes (Vcl, Tmx4, L1cam, Apoc2, Bcl2l2, and Il9r) were selected by HITON-PC as having a causal role in the regression process. Vinculin-1, downregulated in regression, mediates cell adhesion by attaching actin-based microfilaments to the plasma membrane [28]; Tmx4, also down-regulated in regression, is a protein embedded in the endoplasmic reticulum (ER) that potentially facilitates the reduction and folding of yet to be identified client proteins [29]. ApoC2, an upregulated gene in regression, activates lipoprotein lipase [30]. It is important to note that ApoC2 has already been linked to HDL and atherosclerosis [31]. This suggests that the additional factors, although not previously linked to atherosclerosis, are potentially important regulators of regression/progression. Future work will further define their roles as well as the other genes in regression of atherosclerosis.

A study in another mouse model (Reversa mice, a “genetic switch” model in which hyperlipidemia can be conditionally suppressed) [32], found that after lowering plasma lipids at 28 weeks of age, plaque progression slowed over the next 12 weeks, but regression did not occur [33]. Under the conditions of reduced plasma lipid levels, a 37-gene signature was identified in a microarray analysis of aortic homogenates. Only four genes from the Reversa study (Fndc3b, Gypc, Pvrl2 and Stat1) overlapped with the univariate regression gene pattern reported here. This is not surprising since in the Reversa study the lowering of plasma lipids did not result in plaque regression. Further, unlike in the study reported here, in the Reversa study the entire aortic wall, containing a mixture of cell types, rather than laser captured CD68+ cells, was used for mRNA profiling. Thus, the distinct gene expression profiles most likely reflect discrepancies in the extent of lipid lowering to achieve regression, and heterogeneity among the cell types examined.

A limitation of our study is the small amount of total RNA obtained from the laser captured CD68+ cells required a two step amplification process. This procedure amplifies the background noise as well as the biological signal, which is reflected in the high rate of false positives (26%) estimated in the plaque regression differential profile and in some discrepancies observed between qPCR and microarrays (for example in the case of myopalladin and some protocadherins, compare values for these genes in the progression group, Figure 3 with Table S1). Discrepancies between qPCR and microarrays are also seen for two genes identified by local causal pathway discovery (Figures 5A and 5B). Since this discovery method is based on statistical conditioning test of independence that does not consider direction of regulation or effect size, the errors are likely in the side of the microarrays. Nevertheless, we validated our most informative findings by qPCR, demonstrating that within the plaque regression differential profile and the local causal pathway signature there is information with significant biological relevance.

Another limitation of our study is our focus on CD68+ cells. Although the markers indicative of specific sub-populations of monocyte-derived cells (M1 macrophages, M2 macrophages, immature dendritic cells, mature dendritic cells) remains open for debate, it is commonly accepted that CD68 is a marker expressed by all of them. Given that the bulk of monocyte-derived cells in the plaque are macrophages and macrophage-foam cells, we selected CD68 as a convenient criterion to use for laser capture. We focused on macrophages, given their central role in the pathogenesis of atherosclerosis, and the ease of isolation by laser capture microdissection.

We are also aware that that some of the changes in gene expression appear “counterintuitive” in that the levels of “inflammation-associated” factors are increasing rather than decreasing and thus opposite to the overall trend of less inflammation in the regressing plaque. There are a number of potential reasons for this. For example, it is known that the most efficient resolution of inflammation follows a burst of inflammation [34]. Alternatively, because we only selected on one marker (CD68), there could be sub-populations of monocyte-derived cells with divergent inflammatory states. As we progress with our research, for example, by performing FACS analyses with macrophage specific markers of the CD68+ cells isolated from aortic digests, these possibilities will be investigated.

In conclusion, taking advantage of a transplant model of atherosclerosis regression we provide evidence that during regression macrophage mRNA expression is dynamic and that these changes may affect the inflammatory, migratory, and metabolic properties of plaque macrophages. The insights gained from this analysis will deepen the understanding of atherosclerosis regression and could ultimately lead to new therapeutic targets against cardiovascular disease.

## Supporting Information

Table S1 **qPCR assays used to confirm candidate genes.**
(XLS)

Table S2 **The plaque regression differential profile.**
(XLS)

Table S3 **Biological processes enriched in up-regulated and down-regulated genes in the plaque regression signature.**
(XLS)

Table S4 **Genes in selected biological processes.**
(XLS)
